# Recurrent SARS-CoV-2 spike mutations confer growth advantages to select JN.1 sublineages

**DOI:** 10.1080/22221751.2024.2402880

**Published:** 2024-09-11

**Authors:** Qian Wang, Ian A. Mellis, Jerren Ho, Anthony Bowen, Theresa Kowalski-Dobson, Riccardo Valdez, Phinikoula S. Katsamba, Madeline Wu, Caitlin Lee, Lawrence Shapiro, Aubree Gordon, Yicheng Guo, David D. Ho, Lihong Liu

**Affiliations:** a Aaron Diamond AIDS Research Center, Columbia University Vagelos College of Physicians and Surgeons, New York, NY, USA; b Pandemic Research Alliance unit at the Wu Center for Pandemic Research, Columbia University Vagelos College of Physicians and Surgeons, New York, NY, USA; c Department of Pathology and Cell Biology, Columbia University Vagelos College of Physicians and Surgeons, New York, NY, USA; d Division of Infectious Diseases, Department of Medicine, Columbia University Vagelos College of Physicians and Surgeons, New York, NY, USA; e Department of Epidemiology, University of Michigan, Ann Arbor, MI, USA; f Department of Pathology, University of Michigan, Ann Arbor, MI, USA; g Zuckerman Mind Brain Behavior Institute, Columbia University, New York, NY, USA; h Department of Biochemistry and Molecular Biophysics, Vagelos College of Physicians and Surgeons, Columbia University, New York, NY, USA; i Department of Microbiology and Immunology, Columbia University Vagelos College of Physicians and Surgeons, New York, NY, USA; j State Key Laboratory of Virology, College of Life Sciences, Wuhan University, Wuhan, People’s Republic of China; k Taikang Center for Life and Medical Sciences, Wuhan University, Wuhan, People’s Republic of China

**Keywords:** SARS-CoV-2, COVID-19, JN.1, KP.2, mRNA vaccines, antibody evasion, viral receptor binding affinity, antigenicity

## Abstract

The recently dominant SARS-CoV-2 Omicron JN.1 has evolved into multiple sublineages, with recurrent spike mutations R346T, F456L, and T572I, some of which exhibit growth advantages, such as KP.2 and KP.3. We investigated these mutations in JN.1, examining their individual and combined effects on immune evasion, ACE2 receptor affinity, and in vitro infectivity. F456L increased resistance to neutralization by human sera, including those after JN.1 breakthrough infections, and by RBD class-1 monoclonal antibodies, significantly altering JN.1 antigenicity. R346T enhanced ACE2-binding affinity and modestly boosted the infectivity of JN.1 pseudovirus, without a discernible effect on serum neutralization, while T572I slightly bolstered evasion of SD1-directed mAbs against JN.1's ancestor, BA.2, possibly by altering SD1 conformation. Importantly, expanding sublineages such as KP.2 containing R346T, F456L, and V1104L, showed similar neutralization resistance as JN.1 with R346T and F456L, suggesting V1104L does not appreciably affect antibody evasion. Furthermore, the hallmark mutation Q493E in KP.3 significantly reduced ACE2-binding affinity and viral infectivity, without noticeably impacting serum neutralization. Our findings illustrate how certain JN.1 mutations confer growth advantages in the population and could inform the design of the next COVID-19 vaccine booster.

The SARS-CoV-2 Omicron JN.1 subvariant became dominant in early 2024 [1], and prior studies showed that it was already 2–3 times more resistant to serum neutralization than XBB.1.5 [2], resulting in reduced effectiveness of XBB.1.5 monovalent vaccines against JN.1 sublineages [3]. JN.1 has since spawned multiple sublineages, some of which, such as KP.2 and KP.3, have outcompeted the parental JN.1 (Figure 1A). These sublineages contain recurrent spike mutations, including R346 T, F456L, and T572I. KP.2 includes R346 T, F456L, and V1104L, while KP.3 includes F456L, V1104L, and Q493E (Figure 1B and Figure S1). A better understanding of the properties of JN.1 sublineages and their component mutations could explain why they are expanding and aid in the early identification of variants of concern. Of particular interest would be the identification of any sublineages that evade immune responses in individuals previously exposed to JN.1, including KP.2, which the US Food and Drug Administration has recommended as immunogens for the updated COVID-19 mRNA vaccines [4].

**Figure 1.**
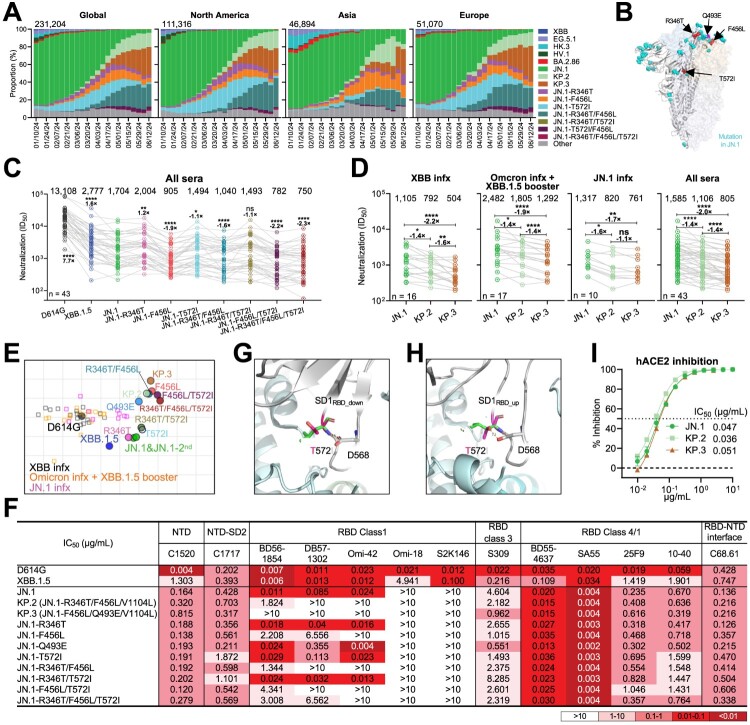
Antibody evasion properties and viral receptor affinities of JN.1 sublineages. (A) Frequency of SARS-CoV-2 variants, including JN.1 sublineages that carry the R346 T, F456L, and T572I mutations. The value in the upper right corner of each box indicates the cumulative number of sequences for all circulating viruses within the specified time period. (B) Key spike mutations of JN.1 and its sublineage in the context of BA.2. JN.1 mutations relative to BA.2 are noted in cyan. Recurrent JN.1 sublineage mutations characterized in this study are noted in red. (C) Serum neutralizing titers (ID_50_) against VSV-based pseudoviruses bearing spike proteins from D614G, XBB.1.5, and SARS-CoV-2 Omicron JN.1-lineage viruses for all samples from three cohorts: “XBB infx”, “Omicron infx + XBB.1.5 booster” and “JN.1 infx” cohorts, as shown in Supplementary information FigureS2a. (D) Neutralizing ID50 titers of serum samples from “XBB infx”, “Omicron infx + XBB.1.5 booster” and “JN.1 infx” cohorts against Omicron JN.1, KP.2 and KP.3. In c and d, the geometric mean ID_50_ titers (GMT) are presented above symbols. The fold change in geometric mean ID_50_ value for each variant compared to JN.1 is also shown above the symbols. Statistical analyses were performed by employing Wilcoxon matched-pairs signed-rank tests. n, sample size. ns, not significant; *p < 0.05; **p < 0.01; ***p < 0.001; ****p < 0.0001. (E) Antigenic map generated using neutralization data from panel B and Figure1A. D614G represents the central reference for all serum cohorts, with the antigenic distances calculated by the average divergence from each variant. One antigenic unit (AU) represents an approximately 2-fold change in ID_50_ titer. Serum samples and viruses are shown as squares and dots, respectively. The data on JN.1 and JN.1-2nd were based on the Fig. a and Supplementary information, FigureS2a, respectively. A shaded area was used to group the viruses bearing the 456L mutation. (F) The antibody concentrations resulting in 50% inhibition of infectivity (IC_50_) are presented. (G) Structural modelling of T572I in BA.2 spike (PDB: 7XIX) in all RBD down conformation. (H) Structure modelling of T572I in RBD up conformation of BA.2 spike (PDB: 7XIW). (I) Sensitivity of pseudotyped JN.1, KP.2, and KP.3 subvariants to hACE2 inhibition. IC_50_ values are also noted. Data are shown as mean ± standard error of mean (SEM) for four technical replicates.

First, we assessed serum virus-neutralizing titers (ID_50_) against key JN.1 sublineages in 43 people across three clinical cohorts: (1) XBB breakthrough infection (XBB infx), (2) Omicron infection followed by XBB monovalent vaccine booster (Omicron infx + XBB.1.5 booster), or (3) JN.1 breakthrough infection (JN.1 infx) (Tables S1 and S2). Additionally, we examined changes in monoclonal antibody (mAb) neutralization of these sublineages using a panel of 13 mAbs targeting multiple known epitopes, aiming to shed light on the how specific mutations confer resistance to specific category of mAbs [5]. Furthermore, we assessed affinity to the human ACE2 (hACE2) using hACE2-inhibition of virus infectivity in Vero-E6 cells as a surrogate.

Consistent with prior results, JN.1 was 1.4-to-1.9 times as evasive of serum antibodies compared to XBB.1.5 (Figure 1C and Figure S2A) [2]. KP.2 and KP.3 exhibited even greater resistance than JN.1, with 1.4-to-2.2 times more evasive to neutralization across the three cohorts, and both were similarly evasive in the JN.1 infx cohort. KP.3 showed slightly more antibody evasion than KP.2 (1.4-to-1.6 fold) in the other two cohorts (Figure 1D). Of all component mutations tested, F456L was the only one associated with substantial increases in evasion of serum neutralization (1.3-to-2.3 times as evasive, Figure S2A). Antigenic cartography demonstrated that sublineages featuring the F456L mutation, regardless of the presence of other mutations, form a distinct cluster with >1 unit more antigenic distance to D614G compared to JN.1 (Figure 1E). F456L also conferred resistance to several RBD Class 1 mAbs (Figure 1F), T572I moderately increased the neutralization sensitivity to soluble human ACE2 and enhanced evasion of SD1 directed mAbs on a BA.2 backbone (Figures S3A and S3B). Structurally, T572I is not located within the epitope of sub-domain 1 (SD1)-directed mAbs, S3H3 and 12-19, as shown in Figure S3C. This suggests that T572I influences the neutralization of these SD-1 antibodies through a conformational alteration of SD-1. Structural modelling indicates that while I572 can still be accommodated on the RBD of BA.2 in its downward conformation (Figure 1G), the T572I mutation disrupts the hydrogen bond between T572 and D568 on the BA.2 RBD in its upward conformation (Figure 1H). This disruption reveals that T572 may affect the dynamics of SD1 during the transition from the RBD down to the RBD up position. Furthermore, R346 T or Q493E mutations did not substantially affect antibody neutralization or serum neutralizing titers (Figure 1F, Figures S2B and S2C). There were no substantial differences in ACE2-inhibition between KP.2, KP.3, and JN.1 (Figure 1I), suggesting no discernible differences in receptor binding, but R346T did enhance more efficient engagement with the ACE2 receptor by ∼1.5-fold and modestly increased viral infectivity (Figure S4). Additionally, other JN.1 sublineages, including KP.3, showed similar infectivity as pseudoviruses in multiple cell lines (Figure S4B). Lastly, the Q493E mutation in KP.3 primarily reduces ACE2-binding affinity and viral infectivity, with minimal impact on serum neutralization (Figure 1F and Figures S2C, S4, and S5).

In summary, we systematically evaluated the antigenic properties of the currently dominant Omicron JN.1 sublineages. Our findings provide valuable insights for vaccine design strategies. Particularly, the immune evasion and growth advantages driven by recurrent mutations in various variants suggest that adaptive immunological responses to these repetitive epitopes in SARS-CoV-2 are under selective evolutionary pressures. To enhance vaccine efficacy, future vaccine components should account for these factors and include key recurrent mutations to elicit more targeted and robust antibody responses against evolving SARS-CoV-2 variants. Furthermore, our study highlights the crucial need to monitor the evolutionary trajectory of these sublineages following the administration of the updated COVID-19 vaccines, based on JN.1 or KP.2, which will be available in the United States starting in Fall 2024.

## Supplementary Material

Supplementary Appendix.docx

## References

[1] Khare S, Gurry C, Freitas L, et al. GISAID's role in pandemic response. China CDC Wkly. 2021;3(49):1049–1051. doi:10.46234/ccdcw2021.255 34934514 PMC8668406

[2] Wang Q, Guo Y, Bowen A, et al. XBB.1.5 monovalent mRNA vaccine booster elicits robust neutralizing antibodies against XBB subvariants and JN.1. Cell Host Microbe. 2024;32(3):315–21. 38377995 10.1016/j.chom.2024.01.014 PMC10948033

[3] Lin DY, Du Y, Xu Y, et al. Durability of XBB.1.5 Vaccines against Omicron Subvariants. N Engl J Med. 2024;390(22):2124–2127. doi:10.1056/NEJMc2402779 38810167 PMC11197991

[4] FDA. Updated COVID-19 vaccines for use in the United States beginning in Fall 2024. 2024 06/13/2024 [cited]; Available from: https://www.fda.gov/vaccines-blood-biologics/updated-covid-19-vaccines-use-united-states-beginning-fall-2024

[5] Wang Q, Guo Y, Liu L, et al. Antigenicity and receptor affinity of SARS-CoV-2 BA.2.86 spike. Nature. 2023;624(7992):639–44. 37871613 10.1038/s41586-023-06750-w

