# Supplementary material for: Recurrent SARS-CoV-2 spike mutations confer growth advantages to select JN.1 sublineages
Source: Emerg Microbes Infect. 2024 Sep 11;13(1):2402880. doi: 10.1080/22221751.2024.2402880 (PMC11407393; doi:10.1080/22221751.2024.2402880)
Supplement: Supplementary Appendix.docx [file TEMI_A_2402880_SM3780.docx]

**Supplementary Appendix**

[Supplementary Figures and Tables 9](#_Toc176081700)

[**Figure S1. Recurrent spike mutations in SARS-CoV-2 Omicron JN.1 sublineages.** 9](#_Toc176081701)

[**Figure S2. Serum neutralization of D614G, XBB.1.5 , and JN.1 sublineages.** 10](#_Toc176081702)

[**Figure S3. Neutralization of BA.2 and BA.2-T572I by ACE2 or mAbs.** 11](#_Toc176081703)

[**Figure S4. Susceptibility to hACE2 inhibition and relative infectivity of the indicated pseudoviruses.** 12](#_Toc176081704)

[**Figure S5. ACE2 affinity of JN.1 spike and JN.1 spikes with individual mutations.** 13](#_Toc176081705)

[**Table S1: Summary of clinical cohorts.** 14](#_Toc176081706)

[**Table S2: Participant demographic, vaccine, and infection details are listed for each sample.** 15](#_Toc176081707)

[Supplementary Methods 16](#_Toc176081708)

[***Clinical Cohorts*** 16](#_Toc176081709)

[***Cell lines*** 16](#_Toc176081710)

[***Plasmid generation*** 16](#_Toc176081711)

[***Protein expression and purification*** 16](#_Toc176081712)

[***Pseudovirus production*** 17](#_Toc176081713)

[***Pseudovirus infectivity*** 17](#_Toc176081714)

[***Pseudovirus neutralization assays with sera, mAbs, or ACE2*** 17](#_Toc176081715)

[***Antigenic cartography*** 18](#_Toc176081716)

[Quantification and statistical analysis 18](#_Toc176081717)

[Author Contributions 18](#_Toc176081718)

[Supplementary References 19](#_Toc176081719)

# **Supplementary Figures and Tables**

## **Figure S1. Recurrent spike mutations in SARS-CoV-2 Omicron JN.1 sublineages.**

Diversification in SARS-CoV-2 Omicron JN.1 sublineages with recurrent R346T, F456L, and T572I mutations up to June 12, 2024.

## **Figure S2. Serum neutralization of D614G, XBB.1.5 , and JN.1 sublineages.**

1. Neutralizing ID_50_ titers of serum samples from “XBB infx”, “Omicron infx+XBB.1.5 booster” and “JN.1 infx” cohorts against D614G, XBB.1.5 , and JN.1 sublingeages.
2. Neutralizing ID_50_ titers of serum samples from “JN.1 infx” cohorts against the indicated SARS-CoV-2 variants, JN.1-R346T/F456L and KP.2.
3. Neutralizing ID_50_ titers of serum samples from “XBB infx”, “Omicron infx+XBB.1.5 booster” and “JN.1 infx” cohorts against JN.1, KP.3 and JN.1-Q493E.

The geometric mean ID_50_ titers (GMT) are presented above symbols. Statistical analyses were performed by employing Wilcoxon matched-pairs signed-rank tests. n, sample size. ns, not significant; *p < 0.05; **p < 0.01; ***p < 0.001; ****p < 0.0001.

## **Figure S3. Neutralization of BA.2 and BA.2-T572I by ACE2 or mAbs.**

1. Sensitivity of pseudotyped BA.2 and BA.2-T572I to hACE2 inhibition. IC_50_ values are also noted. Data are shown as mean ± standard error of mean (SEM) for four technical replicates.
2. Pseudovirus neutralization IC_50_ values for mAbs against BA.2 and BA.2-T572I.
3. T572I is located in the SD1 region of the spike protein and does not directly contact the SD1-directed antibodies S3H3 and 12-19.

## **Figure S4. Susceptibility to hACE2 inhibition and relative infectivity of the indicated pseudoviruses.**

1. Sensitivity of pseudotyped JN.1 sublineages to hACE2 inhibition. IC_50_ values are also denoted. Data are shown as mean ± standard error of mean (SEM) for four technical replicates.
2. Relative infectivity of pseudotyped SARS-CoV-2 JN.1 sublineages compared to the parental virus JN.1 in various target cells. Error bars represent standard error of the mean.

## **Figure S5. ACE2 affinity of JN.1 spike and JN.1 spikes with individual mutations.**

Sensorgrams of the dose-dependent (200, 66.6, 22.2, 7.41, and 2.47 nM) binding curves (red lines) and the fitted data (black lines) of JN.1, JN.1-R346T, JN.1-F456L, JN.1-T572I and JN.1-Q493E spike proteins to human ACE2 (hACE2) tested by surface plasmon resonance (SPR). *K_a_*: association rate constant; *K_d_*: dissociation rate constant; *K_D_*: equilibrium dissociation constant.

## **Table S1: Summary of clinical cohorts.**

## **Table S2: Participant demographic, vaccine, and infection details are listed for each sample.**

Vaccine formulations are denoted as Wild-type (WT), BA.5 Bivalent (BA.5), and XBB.1.5 monovalent (XBB.1.5). Vaccine manufacturers are denoted as Pfizer (P) or Moderna (M).

# **Supplementary Methods**

## ***Clinical Cohorts***

The sera samples were all collected at the University of Michigan through the Immunity-Associated with SARS-CoV-2 Study (IASO) (1). XBB infx and Omicron infx + XBB.1.5 booster cohorts) and the VIVA Study (JN.1 infx cohort), and the collections were conducted under protocols reviewed and approved by the Institutional Review Board of the University of Michigan Medical School. All subjects provided written informed consent. Sera were collected from three cohorts: 1) Individuals who had an XBB sublineage infection (XBB infx) between February and September 2023; 2) Individuals with a prior Omicron sublineage infection between January 2022 and August 2023 followed by an XBB.1.5 monovalent vaccine booster (Omicron infx + XBB.1.5 booster); and 3) Individuals who had a JN.1 sublineage infection (JN.1 infx) in January or February 2024. Details for participants are described in Tables S1 and S2. All serum samples were heat inactivated at 56°C for 30 min before use.

## ***Cell lines***

Vero-E6 (CRL-1586) cells and HEK293T (CRL-3216) cells were obtained from ATCC and cultured at 37°C with 5% CO2 in Dulbecco modified Eagle medium (DMEM) + 10% fetal bovine serum (FBS) + 1% penicillin-streptomycin. Expi293 (A14527) cells were purchased from Thermo Fisher Scientific and maintained in Expi293 expression medium per the manufacturer’s instructions. Vero-E6 cells are derived from African green monkey kidneys. HEK293T cells and Expi293 cells are of human female origin.

## ***Plasmid generation***

As previously described, the antibody sequences for the heavy chain variable (VH) and the light chain variable (VL) domains were synthesized by GenScript, and then cloned into the gWiz vector to produce antibody expression plasmids. For the packaging plasmids for pseudoviruses, mutations were made by using the QuikChange II XL site-directed mutagenesis kit (Agilent) on the JN.1 construct that we previously generated1. For the soluble spike expression plasmids, in addition to the mutations under investigation, the 2P substitutions (K986P, V987P) and a “GSAS” substitution in the furin cleavage site (682-685aa) were introduced in the ectodomain (1-1208aa in WA1) of each of the spikes and then fused with an 8x His-tag at the C-terminus as previously described (2). All constructs were verified using Sanger sequencing prior to use.

## ***Protein expression and purification***

The gWiz-antibody, paH-spike, or pcDNA3-sACE2-WT(732)-IgG1 (Addgene plasmid #154104) plasmid was transfected into Expi293 cells using PEI at a ratio of 1:3, and then the supernatants were collected after five days. The antibodies and human ACE2 (hACE2) fused to a Fc tag were purified with Protein A Sepharose (Cytiva) following the manufacturer’s instructions. For SPR analysis, the hACE2 protein was further purified with Superdex 200 Increase 10/300 GL column. Spike proteins were purified using Ni-NTA resin (Invitrogen) per the manufacturer’s instructions. Molecular weight and purity were confirmed by SDS-PAGE protein electrophoresis prior to use.

## ***Pseudovirus production***

SARS-CoV-2 pseudoviruses were produced in a vesicular stomatitis virus (VSV) background, in which the native VSV glycoprotein was replaced by SARS-CoV-2 spike and its variants, as previously described (3). Briefly, plasmids containing the appropriate spike were transfected into HEK293T cells with PEI. After 24 hours, VSV-G pseudotyped ΔG-luciferase (G*ΔG-luciferase, Kerafast) was added, and then washed with culture medium three times before being cultured in fresh medium for another 24 hours. Anti-VSVG (I1) antibody43 was added to deplete non-pseudotyped viruses. Pseudoviruses were then harvested, centrifuged, and then aliquoted and stored at -80°C.

## ***Pseudovirus infectivity***

Pseudovirus particles bearing various SARS-CoV-2 spike proteins were inoculated onto various cell lines including Vero-E6, Vero-E6-TMPRSS2-T2A-ACE2, 293-ACE2 and Calu3, starting with 50 µl per well in 96-well plates and then subjected to serial dilutions. Following a 16–18-hour incubation at 37°C, we measured the activity of the virus-encoded firefly luciferase in the cell lysates. Subsequently, the luciferase activity for each tested pseudotyped virus, which was not oversaturated, was normalized to the parental D614G S control, with an infectivity set to 1.0.

## ***Pseudovirus neutralization assays with sera, mAbs, or ACE2***

Each SARS-CoV-2 pseudovirus was titrated to standardize viral infectious dose before use in neutralization assays. Serially diluted (seven dilutions of) heat-inactivated sera or antibodies were added in 96-well plates, starting at 1:100 dilution for sera and 10 µg/mL for antibodies. For ACE2 inhibition assays, as previously reported, we used soluble chimeric human ACE2, which contains ACE2 residues 1-732 fused to human IgG1 Fc. hACE2 was diluted starting from 10 µg/mL with a dilution factor of two across 11 serial dilutions. Then, pseudoviruses were added and incubated at 37 °C for 1 hour. In each plate, wells containing only pseudoviruses were included as controls. Vero-E6 cells were then added at a density of 4 × 10^4^ cells per well and incubate at 37 °C for an additional 16 hours. Cells were lysed and luminescence was determined by the Luciferase Assay System (Promega) and SoftMax Pro v.7.0.2 (Molecular Devices) according to the manufacturers’ instructions. Data were analyzed in GraphPad Prism v.9.3.

## ***Antigenic cartography***

Antigenic distances between sera, D614G, XBB.1.5, JN.1 and other SARS-CoV-2 JN.1 sublineages were determined by integrating all ID_50_ values of individual serum samples through a published antigenic cartography approach (4). The visualization was generated using Racmacs (v.1.1.4, https://acorg.github.io/Racmacs/) in R version 4.0.3. The optimization step count was set at 2,000 and the minimum column basis parameter set to ‘none’, the ‘mapDistances’ function was employed to calculate antigenic distances between each serum sample and variant.

# **Quantification and statistical analysis**

Neutralization ID_50_ and IC_50_ values were determined by fitting a five-parameter dose-response curve in GraphPad Prism v9.3. Statistical significance of differences in neutralizing titer was evaluated using two-tailed Wilcoxon matched-pairs signed-rank tests in GraphPad Prism v9.3. Significance is presented as following: ns, not significant; *p < 0.05; **p < 0.01; and ***p < 0.001, and ****p < 0.0001.

# **Author Contributions**

L.L., A.G., and D.D.H. conceived and supervised the project. Q.W. managed the project. Q.W. and L.L. constructed the spike expression plasmids. Q.W., I.A.M., J.H., and L.L. conducted pseudovirus neutralization assays. Q.W., J.H., and L.L. purified SARS-CoV-2 soluble spike proteins, hACE2 protein and monoclonal antibodies. Y.G. conducted bioinformatic analyses. C.G., R.V., A.G., and A.B., provided clinical samples and organized information. Q.W., I.A.M., Y.G., A.B., P.S.K., L.S. L. L., and D.D.H. analyzed the results and wrote the manuscript. All authors have reviewed the results and have given their approval for the final version of the manuscript.

# **Supplementary References**

1. Simon V, Kota V, Bloomquist RF, Hanley HB, Forgacs D, Pahwa S, et al. PARIS and SPARTA: Finding the Achilles' Heel of SARS-CoV-2. mSphere. 2022 Jun 29;7(3):e0017922.

2. Wrapp D, Wang N, Corbett KS, Goldsmith JA, Hsieh CL, Abiona O, et al. Cryo-EM structure of the 2019-nCoV spike in the prefusion conformation. Science. 2020 Mar 13;367(6483):1260-3.

3. Liu L, Wang P, Nair MS, Yu J, Rapp M, Wang Q, et al. Potent neutralizing antibodies against multiple epitopes on SARS-CoV-2 spike. Nature. 2020 Aug;584(7821):450-6.

4. Smith DJ, Lapedes AS, de Jong JC, Bestebroer TM, Rimmelzwaan GF, Osterhaus AD, et al. Mapping the antigenic and genetic evolution of influenza virus. Science. 2004 Jul 16;305(5682):371-6.
